# Impact of water hardness on oxytetracycline oral bioavailability in fed and fasted piglets

**DOI:** 10.1002/vms3.185

**Published:** 2019-07-08

**Authors:** Julieta M. Decundo, Susana N. Diéguez, Guadalupe Martínez, Agustina Romanelli, María B. Fernández Paggi, Denisa S. Pérez Gaudio, Fabián A. Amanto, Alejandro L. Soraci

**Affiliations:** ^1^ Área Toxicología Departamento de Fisiopatología Facultad de Ciencias Veterinarias Universidad Nacional del Centro de la Provincia de Buenos Aires Tandil Argentina; ^2^ Facultad de Ciencias Veterinarias Centro de Investigación Veterinaria de Tandil (CIVETAN) UNCPBA‐CICPBA‐CONICET Tandil Argentina; ^3^ Comisión de Investigaciones Científicas de la Provincia de Buenos Aires (CIC‐PBA) Tandil Argentina; ^4^ Área Fisiología de la Nutrición Departamento de Fisiopatología Facultad de Ciencias Veterinarias Universidad Nacional del Centro de la Provincia de Buenos Aires Tandil Argentina; ^5^ Área Producción Porcina Departamento de Producción Animal Facultad de Ciencias Veterinarias Universidad Nacional del Centro de la Provincia de Buenos Aires Tandil Argentina

**Keywords:** bioavailability, oxytetracycline, piglets, water hardness

## Abstract

Water hardness is a critical factor that affects oxytetracycline dissolution by chelation with cations. These interactions may lead to impaired dosing and consequently decrease absorption. Moreover, feed present in gastrointestinal tract may interact with antibiotic and alter pharmacokinetic parameters. In the present study, dissolution profiles of an oxytetracycline veterinary formulation were assessed in purified, soft and hard water. Furthermore, oxytetracycline absolute bioavailability, after oral administration of the drug dissolved in soft or hard water, was evaluated in fed and fasted piglets. A maximum dissolution of 86% and 80% was obtained in soft and hard water, respectively, while in purified water dissolution was complete. Results from in vivo study reconfirmed oxytetracycline's very low oral bioavailability. The greatest values were attained when antibiotic was dissolved in soft water and in fasted animals. Statistically significant lower absolute bioavailability was achieved when hard water was used and/or animals were fed. Moreover, Cmax attained in all treatments was lower than MIC90 of most important swine pathogens. For these reasons, the oral use of OTC formulations, that have demonstrated low oral bioavailability, should be avoided to treat systemic diseases in pigs.

## Abstract 2

In the present study, dissolution profiles of an oxytetracycline veterinary formulation were assessed in purified, soft and hard water. Furthermore, oxytetracycline absolute bioavailability, after oral administration of the drug dissolved in soft or hard water, was evaluated in fed and fasted piglets. A maximum dissolution of 86% and 80% was obtained in soft and hard water respectively, while in purified water dissolution was complete. Results from in vivo study reconfirmed oxytetracycline's very low oral bioavailability, the lowest value statistically significant was achieved when hard water was used and/or fed animals. The use of low oral bioavailability antibiotics represents a risk factor that might lead to therapeutic failure and antimicrobial resistance, for these reasons the oral use of oxytetracycline to treat systemic diseases in pig production should be avoided.

## Graphical abstract

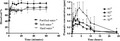

## 1 INTRODUCTION

Feed and water are widely used vehicles for antibiotic administration in intensive pig farming. The possibility of providing therapy to a large number of animals minimizing labour requirements are known benefits over injectable use. However, after oral administration, drugs might not be properly absorbed leading to therapeutic failures, increased bacterial resistance and antibiotic residues accumulation in the environment (Cromwell, 2002; de Souza & Hidalgo, 1997; Wegener, 2003). Medication through feed is generally the preferred option in pig farms due to its relative simplicity; nonetheless, water medication provides important advantages making it the most recommended option for therapeutic management (Soraci, Amanto, Tapia, De la Torre, & Toutain, 2014).

Automatic water proportioning systems (such as Dosatron^®^) are used worldwide in intensive pig production farms. These devises depend on a pump to deliver specific amounts of drugs, dissolved in a stock solution, into the water line at a typical dilution of 1% to 5%. Physicochemical characteristics of the water in the stock solution influence dissolution and stability of drugs. Besides, water quality is highly important as it has a great impact on animal performance and production profits (Burch, 2013; Gustave, 2010). Among water quality factors, hardness is a critical aspect when automatic delivery systems are used to administer certain antibiotics which are prone to interact with cations forming relatively stable complexes (Olkowski, 2009). The most relevant examples are tetracyclines which may be chelated by cations present in water decreasing dissolution and/or further absorption (Bagheri Gh, 2015; Doluisio & Martin, 1963; Hundt et al., 2016; Lambs, Brion, & Berthon, 1984).

Moreover, it has been demonstrated that food present in the alimentary tract directly influences pharmacokinetic processes of many drugs (Abuhelwa, Williams, Upton, & Foster, 2017; Gura, 2016; Welling, 1989). Even if drugs are dissolved in drinking water, they may interact with feed compounds present in the gut. Nielsen and Gyrd‐Hansen (1996) have shown that fasting increases tetracyclines’ bioavailability in pigs. From a practical point of view, this is of major relevance as most bioavailability studies are performed in fasted animals while in intensive production animals are never fasted before antibiotic administration.

Oxytetracycline (OTC) is a classic broad spectrum antibiotic used in pig farming to treat respiratory and gastrointestinal diseases. In spite of its low oral bioavailability (between 5% and 15%) and relatively high MIC values due to antimicrobial resistance development (del Castillo, Elsener, & Martineau, 1998; Kilroy, Hall, Bane, Bevill, & Koritz, 1990; Mevius, Vellenga, Breukink, Nouws, & Vree, 1986; Nielsen & Gyrd‐Hansen, 1996; Pijpers, Schoevers, Haagsma, & Verheijden, 1991), it is authorized worldwide to be administered via feed or water to treat systemic diseases in intensive pig production. Its low cost, and the limited availability of antibacterial agents make OTC to be frequently used in many countries (Larsen et al., 2016; Papich, 2016).

To our knowledge, there are no studies that consider the impact of drinking water hardness and feed present in the gastrointestinal tract on this antibiotic bioavailability. These interactions would be most relevant at weaning when absorption process at intestine level is slowed down as a consequence of stress syndrome that directly affects intestinal mucosa (Campbell, Crenshaw, & Polo, 2013; Heo et al., 2013; Spreeuwenberg, Verdonk, Gaskins, & Verstegen, 2001). On the other hand, weaning is the phase of pig rearing where antibiotics are mainly used (Collineau et al., 2014; Hémonic, Chauvin, & Corrégé, 2014).

The aim of the present study was to evaluate the impact of drinking water hardness and food present in gastrointestinal tract (GIT) on OTC bioavailability after oral administration to weaning piglets.

## 2 MATERIALS AND METHODS

### 2.1 Chemicals and reagents

For oral administration and dissolution assay, Oxytetracycline 50% powder formulation was used (provided by Bedson Laboratories Argentina). For intravenous administration, oxytetracycline hydrochloride veterinary solution for injections (Terramicina^®^, Zoetis, Argentina) was used.

Oxytetracycline chloride analytical standard (97% purity) and doxycycline (Internal Standard, IS) were supplied by Sigma Aldrich, Mo (USA). HPLC grade acetonitrile and methanol were provided by Sintorgan S.A, Argentina. Purified, deionized water was obtained by water purification equipment Pure Lab UHQ de ELGA (Lane End, UK).

### 2.2 Dissolution assay

Dissolution efficiency of a given compound may greatly affect its absorption after oral administration. An in vitro study was performed in order to evaluate dissolution efficiency and stability of an OTC formulation for veterinary use in a simulated stock solution of an automatic delivery system. Dissolution assays were performed following USP 40 (2017) (using apparatus II, paddle) with some modifications. Antibiotic concentration of this solution was calculated in order to carry out a reduced scale model of a commercial farm situation.

Purified water, considered reference (pH: 6), soft and hard water, prepared by adding 40 mg/L CaCO3 (pH 7.3) and 400 mg/L CaCO3 (pH 9), respectively, were used for this assay (EMEA Committee for Medicinal Products for Veterinary use (CVMP), 2005). These represent typical water hardness values found in swine production areas in Argentina (Bagley, Kotuby‐Amacher, & Farrel‐Poe, 1997; Bavera, 2001; Cirelli, Schenone, Pérez Carrera, & Volpedo, 2010).

In order to mimic the way stock solutions are prepared in the farm, the antibiotic was incorporated at once to the recipient containing water. From this moment, considered time 0, sampling times were 0,5; 1; 1,5; 2; 3; 6; 9; 12; 15; 20; 30; 40; 50; 60 min in order to obtain dissolution profiles. For stability evaluation, further sampling points were taken up to 24 hr. For OTC quantification, samples were immediately filtered through a 0.22‐μm nylon membrane and diluted with mobile phase before injection into the HPLC system. Dissolution percentage was calculated using the following equation:

$$\text{Dissolved}\;\%=\frac{\text{A}}{\text{B}}\times 100$$

A: Peak area of the test solution.

B: Peak area of standard solution.

According to USP 40 (2017), dissolution is considered adequate when 80% of the drug are dissolved within 60 min. All dissolution tests were performed in quadruplicates. Maximum dissolution time was calculated as an average between time points where maximum concentration was obtained for different repeats.

#### 2.2.1 In vivo study

In vivo studies were carried out in an intensive pig production farm in Tandil, Buenos Aires Province, Argentina. All animals were treated and handled according to Guidelines of Animal Welfare Committee of the University of the Center of Buenos Aires Province and the experimental design of this study was evaluated and approved according to ethical statement Animal Welfare Committee FCV‐UNCPBA (Res Nº 087/02).

### 2.3 Animals

Sixteen weaning piglets, 30–32 days old, clinically healthy (12 ± 2 Kg body weight) and from the same commercial genetic line were used in this study. Animals were housed in an environmentally controlled barn and were given free access to water and feed (receiving weaning commercial feed out of fasting periods). Mineral concentrations present in feed were analysed by atomic absorption spectrometry (Laboratory of Biochemical Analysis and Minerals, FCV‐UNCPBA) (Table 1).

**Table 1:** Mineral concentrations (Mean and *SD*) of commercial feed sample expressed as dry matter % (DM %) for calcium (Ca), magnesium (Mg) and phosphorous (P) and as ppm of dry matter (ppm DM) for copper (Cu) and zinc (Zn)

Sample	Minerals	Mean	*SD*
Commercial feed	[Ca] (DM %)	1.07	0.213
	[Mg] (DM %)	0.18	0.009
	[P] (DM %)	0.36	0.020
	[Cu] (ppm DM)	153.27	7.255
	[Zn] (ppm DM)	531.83	16.364

The day before treatments, the animals were sedated with diazepam‐ketamine at 2 mg/kg and 15 mg/kg, respectively, and a permanent catheter was placed in the left external jugular vein according to the method proposed by Soraci et al. (2010); the catheter was maintained permeable with heparin solution. This technique facilitates blood sampling and reduces stress in piglets.

### 2.4 Experimental design

The minimum number of animals required for the study was determined using the resource equation (Festing, 2011; Mead, 1988). The experiment was performed following a 2 × 2 factorial design where one factor was fed condition (fasted/fed) and another factor was water hardness (soft/hard). Animals were divided in four groups: Two groups received OTC oral formulation at 40 mg/Kg body weight in soft water (CaCO_3_ 40 mg/L, pH: 7.3) after overnight fasting (S1; *n* = 4) or without fasting (S2; *n* = 4), and two groups received the same treatment in hard water (CaCO_3_ 400 mg/L, pH: 9) in fasted (H1; *n* = 4) or fed (H2; *n* = 4) state. All animals received an IV dose (20 mg/kg body weight OTC) for absolute bioavailability calculation. A 53 hr washout period (corresponding to 10 half‐life) was left between treatments (del Castillo et al., 1998; Grabowski, Marczak, & Okoniewska, 2016).

Blood samples (1.5 ml each) were collected at 0, 5, 10, 15, 30, 45 min; 1, 2, 3, 4, 6, 8, 10, 12 and 24 hr after IV administration and at 0, 15, 30 min; 1, 2, 3, 4, 6, 8, 10, 12 and 24 hr after oral dosage. They were immediately centrifuged at 1,500*g* for 15 min and the supernatant (plasma) was recovered, identified and stored at −20°C until analysis.

For intravenous administration, antibiotic was delivered as bolus through a catheter (Abbocath^®^) sited in the right external jugular vein. For oral administration, the entire antibiotic dose was dissolved in the corresponding water (hard or soft) incorporated through oral gavage.

After treatments, animals were supplied with the same water used for antibiotic delivery and received feed ad libitum. Feed and water consumption, as well as animals′ health were monitored all along the study by qualified personnel. Any changes in behaviour feed or water intake and abnormal situations (vomiting, diarrhoea, excitability, etc.) were registered.

### 2.5 Sample processing

A mixture of 450 μl of each plasma sample, 50‐μl internal standard doxycycline (IS) 40 μg/ml dissolved in mobile phase and 1‐ml acetonitrile was vortexed for 1 min and then centrifuged at 11,200 *g* for 10 min (Gelec^®^ Argentina) in order to separate proteins. The supernatant was evaporated under air flow in a Turbo Vap workstation (Massachusetts, USA) and resuspended in 3‐ml McIlvaine‐EDTA solution prepared according to Miller, Reimschuessel, and Carson (2007). Strata^®^ polymeric reversed phase (33 μm) 200 mg/3 ml cartridges (Phenomenex) were used for solid phase extraction under vacuum (Visiprep Supelco Vacuum Manifold) according to Fritz and Zuo (2007). OTC and IS were eluted with 2 ml of HPLC grade methanol at 1 ml/min flow rate. Eluate was evaporated under stream of nitrogen, resuspended in 500 μl of mobile phase and filtered through a 0.22‐μm nylon membrane before injection into HPLC system.

### 2.6 Chromatographic conditions

The HPLC consisted on a Gilson binary pump system equipped with Gilson 151 UV‐Vis detector and automatic injector (Thermo Scientific UltiMate 3000). Separation was achieved on an ODS Luna C18 of 5 μm, 250 × 460 mm (Phenomenex) column maintained at 40°C. The mobile phase consisted on: A (acetonitrile: methanol 1:1) and B (oxalic acid 0.01 M) 1:1 working in isocratic mode, at 1.5 ml/min. Sample injection volume was 50 μl and chromatographic run time was 5 min. OTC and the IS were detected at 365 nm and retention times were 1.9 and 2.5 min respectively.

Quantification was carried out using the ratio between oxytetracycline and its IS doxycycline as the assay response. Validation parameters, as well as their acceptance range, were in accordance with international guidelines (U.S. Department of Health and Human, Food and Drug Administration FDA, CDER, CVM. Guidance for Industry, Bioanalytical Method Validation, 2018).

Calibration curves were prepared in triplicates, and assayed within 1 week, in order to assess linearity. Least square linear regression was used for curve fitting.

Quality control samples fortified at three levels were processed in triplicates on four separate days, in order to assess accuracy and precision of the method. The accuracy was expressed as relative error (RE) and it was required to be ±15% (except for the limit of detection where it could reach up to 20%). Within‐day precision (repeatability) was calculated in terms of mean coefficient of variation (CV) that was required to be less than 15% for all concentrations (except for the limit of detection where it could reach up to 20%). Between day precision (intermediate precision) was expressed as between day coefficient of variation, which was calculated using the following equation:

$${\text{CV}}_{\mathrm{bd}}=\frac{SD_{\mathrm{bd}}}{\mu }$$

Being:

*μ*: average media.

*SD*_bd_: between day standard deviation (calculated as the square root of between days variance).

Between day variance was obtained after subtracting the contribution of within day variability, using the following equation:

$$SD_{\mathrm{bd}}^{2}=SD^{2}\left(\mu \right)+\frac{n-1}{n}SD_{\mathrm{wd}}^{2}$$

Being:

*SD* ^2^(*μ*): variance of every day mean.

*n*: number of observations per day.

*SD* ^2^_wd_: average within day variance.

Lower limit of quantification was defined as the lowest concentration at which both precision and accuracy were less than or equal to 20%, and it was obtained by analysing the fortified plasma at the lower level of the calibration curve in five replicates on three different days.

### 2.7 Data analysis

For in vitro study, Kruskal‐Wallis test was performed to compare results followed by Dunn's test which was used to determine differences among groups using R Studio software version 1.1.456 (RStudio Team, 2015).

Pharmacokinetic (PK) parameters, maximum concentration (*C*_max_), area under the concentration‐time curve (AUC_0–24_), time at which *C*_max_ is reached (*T*_max_) and absolute bioavailability (*F*%), were estimated for each animal. Results were expressed as mean ± standard deviation for each group. The analysis was carried out following non‐compartmental method based on statistical moment (Gibaldi & Perrier, 2007) using PK Solutions 2.0 software (Farrier, 1997).

AUC_0–24_ for oxytetracycline was estimated by trapezoidal method and F% was calculated according to the following equation (Gibaldi & Perrier, 2007; Toutain & Bousquet‐Mélou, 2004):

$$F\%=\frac{{\text{AUC}}_{\mathrm{PO}}}{{\text{AUC}}_{\mathrm{IV}}}\times \frac{{\text{Dose}}_{\mathrm{IV}}}{{\text{Dose}}_{\mathrm{PO}}}\times 100$$

AUC_PO_ is the area under the concentration‐time curve for oral administration.

AUC_IV_ is the area under the concentration‐time curve for intravenous administration.

Dose_PO_ and Dose_IV_ are oral and intravenous doses respectively.

Pharmacokinetic parameters were compared by two‐way analysis of variance (ANOVA) or Kruskal‐Wallis test when normality was not met. Differences among groups were detected by Tukey's test. All statistical analysis was performed using the software RStudio version 1.1.456 (RStudio, T, 2015). A *p* value of 0.05 was considered to denote significant differences.

## 3 RESULTS

### 3.1 Method validation

Good linearity was obtained within the concentration range, being r^2^ coefficient above 0.997 for all replicates.

Accuracy and precision were evaluated for spiked samples at 1, 2 and 4 μg/ml. Accuracy, expressed as relative error, was −12.5%, 2% and −6% respectively. Repeatability (Within‐day precision) and intermediate precision (between day precision) were less than 10% for all concentrations studied. Lower limit of quantification (LOQ) was 0.125 μg/ml.

### 3.2 Dissolution assay

**Figure 1:**
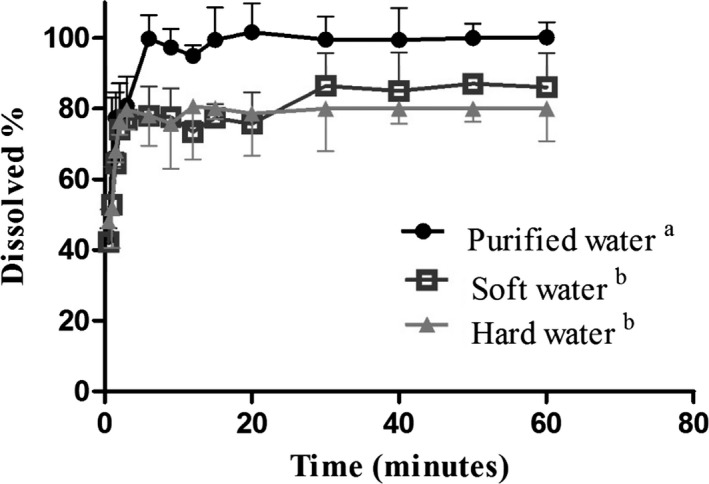
Dissolution profiles of OTC formulation in purified, soft water and hard water. Different superscript letters indicate significant differences among dissolution profiles (*p* < 0.05)

Figure 1 shows dissolution profiles of the OTC formulation in water with different characteristics. OTC formulation in purified water (reference) achieved 100% dissolution in 3 min. A maximum dissolution of 86% in 25 min and 80% in 13.5 min was obtained for soft and hard water respectively. Statistically significant differences were determined between purified water and soft or hard water (*p* < 0.05).

#### 3.2.1 In vivo study

All animals kept clinically healthy and feed and water (hard or soft water) intake were normal all along the assay. No side effects of treatments were observed.

Table 2 lists relevant PK parameters (mean ± *SD*) for each group. No statistically significant differences were observed for *T*_max_ values between treatments.

**Table 2:** Pharmacokinetic parameters (mean ± *SD*) of 40 mg/kg body weight oral OTC administered in soft water after overnight fasting (S1), without fasting (S2), in hard water after fasting (H1) and without fasting (H2)

Pharmacokinetic parameters	**S1**(*n* = 4) Mean ± *SD*	**S2** (*n* = 4) Mean ± *SD*	**H1** (*n* = 4) Mean ± *SD*	**H2** (*n* = 4) Mean ± *SD*
*C*_max_ (μg/ml)^b^	0.77 ± 0.19^a^	0.52 ± 0.36^a^, ^b^	0.47 ± 0.13^a^, ^b^	0.30 ± 0.08^b^
*T*_max_ (h)^a^	4.00 ± 1.41	2.75 ± 1.50	3.5 ± 2.08	2.5 ± 1.00
AUC _0–24_ μg·hr ml^−1^)^b^	6.27 ± 2.04^a^	3.72 ± 1.50^a^, ^b^	3.15 ± 0.89^b^	2.05 ± 0.29^b^
*F*%^b^	6.13 ± 1.99^a^	3.64 ± 1.46^a^, ^b^	2.83 ± 0.44^b^	2.00 ± 0.28^b^

Abbreviations: AUC_0–24_, area under the concentration‐time curve from 0 to 24 hr; *C*_max_, maximum plasma concentration; *F*%, absolute bioavailability; *T*_max_, time of maximum concentration.

a No significant differences were observed between the different groups (*p *> 0.05).

b Different superscript letters indicate significant differences among groups (*p* < 0.05).

S1 exhibited the highest Cmax (0.77 ± 0.19) and *F*% (6.13 ± 1.99) values. Statistical significant differences for Cmax were obtained between S1 and H2 (*p* < 0.05). S1 absolute *F*% was significantly higher than H1 (*p* < 0.05) and H2 (*p* < 0.05).

**Figure 2:**
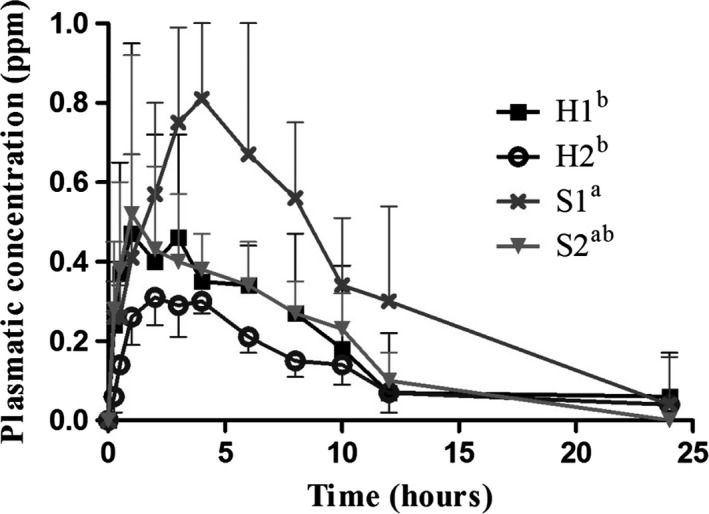
OTC mean plasma levels after 40 mg/kg body weight dose administered in soft water after overnight fasting (S1, *n* = 4), without fasting (S2, *n* = 4), in hard water after fasting (H1, *n* = 4) and without fasting (H2, *n* = 4). Different superscript letters indicate significant differences among AUC_0–24_ of the different groups (*p* < 0.05)

Figure 2 shows plasma concentration—time curves from which AUC_0–24_ was obtained. Considerable variation was observed within each group. Group S1 showed the highest values (6.27 ± 2.04) which were significantly different from H1 (*p* < 0.05) and from H2 (*p* < 0.05).

## 4 DISCUSSION

When performing an oral antibiotic therapy through water in swine production, the dissolution of the formulation represents an important variable that, together with the voluntary consumption, has a strong impact on the dosage (Pijpers et al., 1991; Soraci et al., 2014).

A correct antibiotic dissolution depends on physicochemical properties of the formulation and water quality. Hardness of drinking water is an important aspect to be considered because it could interfere with drug dissolution (Gustave, 2010; Hörter & Dressman, 2001; Jambhekar & Breen, 2013; Mcleese, Tremblay, Patience, & Christison, 1992; Sugano & Terada, 2015).

In the present study, in vitro dissolution of OTC formulation in soft and hard water showed a decrease in 14% and 20%, respectively, compared to 100% dissolution of the same formulation in purified water. The specific interaction between OTC and calcium generates partially insoluble chelation complexes (Brion, Berthon, & Fourtillan, 1981; Brion, Lambs, & Berthon, 1985; Martin, 1979; (Novak‐Pekli, el‐Hadi Mesbah, & Petho, 1996). At different pH values, electron donor functional groups of OTC can suffer protonation or deprotonation and then can be stabilized by chelation with the positive charge of metal ions like calcium and magnesium (Cesaretti et al., 2014; Katlam, Deshmukh, & Jadhav, 2017; Schmitt & Schneider, 2000; Werner, 2006). The affinity between divalent cations and OTC molecule increases with pH (Pulicharla, Hegde, Kaur, & Surampalli, 2017). In this way, the use of hard water, with high pH, to dissolve the OTC formulation, renders lower antibiotic concentrations in the medicated water. Around the world, pig production farms are often located in areas where water hardness is high. This is frequently an overlooked aspect in swine veterinary practices which directly affects antibiotic dosage leading to therapeutic failures and increased bacterial resistance. Therefore, guaranteeing OTC formulation quality and its dissolution in drinking water or stock solution of automatic delivery systems should be a main goal of veterinary pharmaceutical industry.

OTC bioavailability was low in all treatment groups with high inter‐individual variability. This is in agreement with previous studies in pigs (Kilroy et al., 1990; Mevius et al., 1986; Nielsen & Gyrd‐Hansen, 1996; Pijpers et al., 1991; Welling, Koch, Lau, & Craig, 1977). In addition, when OTC was administered to fasted animals in our study, *F*% and AUC_0–24_ of the antibiotic dissolved in hard water was around 50% lower than *F*% and AUC_0–24_ of the antibiotic dissolved in soft water (*p* < 0.05). Similar results were obtained by Ziółkowski et al. (2016) after administration of OTC in water containing different concentrations of divalent cations to broiler chickens. Results obtained in both studies could be related to changes in conformational structure of chelated OTC that affect its interaction with mucosal cells’ membrane decreasing absorption, hence AUC_0–24_ and *F*%. (Cesaretti et al., 2014; Lunestad & Goksayr, 1990; Nelson, 1998; Neuvonen, 1976; Pulicharla et al., 2017; Schmitt & Schneider, 2000).

As a direct consequence, under‐dosing and lack of reproducibility in terms of clinical efficacy would be expected (Toutain & Bousquet‐Mélou, 2004), promoting antimicrobial resistance and drug accumulation in the environment (Gamsiz et al., 2010; Wegener, 2003).

When OTC was dissolved in soft water, animals receiving treatment in fed state showed 41% lower *F*% than fasted animals. Even though these values were not statistically significant (high inter‐individual variations must be considered and a much larger sample size might be necessary to detect statistical differences), the presence of calcium and other cations present in enteral feed could interact with OTC modifying PK absorption parameters. In fact, Wanner, Nietlispach, and Sutter (1990); Wanner, Walker, Sutter, Riond, and Broz (1991) demonstrated that molecules present in feed can decrease (Ca ions) or enhance (citric acid) tetracyclines’ bioavailability in pigs. Other authors described similar interactions with different feed components (Magnam, Barthes, & Giraud, 1986; Mapp & McCarthy, 1976; Pérez et al., 2018). When hard water was used to dissolve the antibiotic formulation, no differences were observed in PK parameters from fed or fasted animals. It could be hypothesized that the effects of ions in hard water solution overtake the effect of ions from feed. Our results, when hard water is used to administer the antibiotic, are in agreement with the work of Nielsen and Gyrd‐Hansen (1996) who studied OTC *F*% in fasted and fed piglets and found no statistically significant differences, being 3 ± 1% in both cases, but the authors do not describe characteristics of the water used in the study. Together with low bioavailability in our study, Cmax in all treatment groups fell below MIC90 of OTC reported for most important swine pathogens; namely 1 μg/ml for *H.parasuis*, 2 μg/ml for *A. pleuropneumoniae,* 16 μg/ml for *P. multocida* and 64 μg/ml for *B. bronchiseptica* and *S. suis* (Dorey, Pelligand, Cheng, & Lees, 2017; de Jong et al., 2014). *C*_max_ values were higher in fasted than in fed state for both water qualities; however, these results were similar to those of Nielsen and Gyrd‐Hansen (1996) where *C*_max_ was 0.7 ± 0.3 μg/ml in fasting and 0.4 ± 0.1 μg/ml without fasting. The same tendency was maintained with AUC_0–24_ results. Besides, there were no statistically significant differences for *T*_max_ in both studies. By contrast, *C*_max_ values reported by Pijpers et al. (1991) were higher (1.87 ± 0.29 μg/ml) than the values obtained in the present study, but the research was conducted at higher doses (50 mg/Kg) in growing‐finishing pigs. Similar results were obtained by Mevius et al. (1986) in spite of using a lower dose (20 mg/Kg) in post weaning pigs. These discrepancies are expected from oral use of molecules that are poorly absorbed (Toutain & Bousquet‐Mélou, 2004). In this scenario, results must be compared thoughtfully, as they are very much influenced by the age of the animals, stress, physiological state of gastrointestinal tract, diet, route of administration, pharmaceutical formulation, etc. High variability of results has been described between, and even within, different assays.

Results from the present study reconfirm the very low OTC oral bioavailability. Interactions with cations present in drinking water deepen this situation. Water quality is an important aspect to be considered in order to attain complete OTC dissolution and assure correct dosage. In any case, the oral use of low bioavailability antibiotics in intensive animal production that do not get over MIC_90,_ represents a crucial risk factor for antimicrobial resistance development and therapeutic failures. Microbiota in caecum and colon is exposed to high concentrations of unabsorbed fraction of the OTC administered (>90%) (Hansen, Aarestrup, & Sørensen, 2002; Toutain, Ferran, Bousquet‐Melou, Pelligand, & Lees, 2016), enhancing multidrug‐resistant strains (Herrick, Haynes, Heringa, Brooks, & Sobota, 2014; Toutain et al., 2016) and drug dissemination in the environment (Cheng et al., 2014). Accounting for the seriousness of this situation and in a context where rational use of antibiotics is essential, we think that if low oral bioavailability has been demonstrated for a certain formulation, as it is the case of OTC in the present study, its oral use should not be acceptable to treat systemic infections.

## ACKNOWLEDGEMENTS

The authors thank Edgardo Rodriguez for collaborating with statistical design and Valeria Eguia for collaborating with laboratory work.

## CONFLICT OF INTEREST

No potential conflict of interest was reported by the authors.

## SOURCE OF FUNDING

This study was funded by Centro de Investigación Veterinaria de Tandil (CIVETAN) UNCPBA‐CICPBACONICET, TandilArgentina.

## ETHICAL STATEMENT

The authors confirm that the ethical policies of the journal, as noted on the journal's author guidelines page, have been adhered to and the appropriate ethical review committee approval has been received. Guidelines of Animal Welfare Committee of the University of the Center of Buenos Aires Province were followed.

## CONTRIBUTIONS

Julieta M. Decundo: experimental design, surgery assistance, in‐farm work and animal handling, sample collection, in vitro study, analytical procedure, results of pharmacokinetic and statistical analysis, and paper writing; Susana N. Diéguez: analytical method development and validation, analytical procedure, pharmacokinetic and statistical analysis results analysis and discussion, and paper writing; Guadalupe Martinez: collaboration in surgery, in‐farm work and animal handling and sampling. Results’ discussion; Agustina Romanelli: in‐farm work and animal handling, sample collection; María B. Fernández Paggi: collaboration in‐farm work and animal handling and sampling; Denisa. S. Pérez Gaudio: analysis and discussion of pharmacokinetic results; Fabian A. Amanto: in‐farm work, animal handling and sample collection; Alejandro L. Soraci: Project Director, experimental design, animal surgery, in‐farm work and animal handling, analytical procedure, statistical analysis results analysis and discussion and paper writing and correction.
